# Imagining a future in global health without visa and passport inequities

**DOI:** 10.1371/journal.pgph.0002310

**Published:** 2023-08-23

**Authors:** Shashika Bandara, Zahra Zeinali, (Dian) Maria Blandina, Omid V. Ebrahimi, Mohammad Yasir Essar, Joyeuse Senga, Mehr Muhammad Adeel Riaz, Iwatutu Joyce Adewole, Marie-Claire Wangari

**Affiliations:** 1 Faculty of Medicine and Health Sciences, Department of Family Medicine, McGill University, Montreal, Canada; 2 Department of Global Health, University of Washington, Seattle, Washington, United States of America; 3 Laboratory of Primary Health Care, General Medicine, and Health Services Research, Aristotle University of Thessaloniki, Thessaloniki, Greece; 4 People’s Health Movement, Global Health Governance Program; 5 University of Oslo, Department of Psychology, Oslo, Norway; 6 Modum Bad Psychiatric Hospital and Research Center, Vikersund, Norway; 7 Department of Global Health, McMaster University, Hamilton, Ontario, Canada; 8 Department of Epidemiology, Biostatistics, and Occupational Health, School of Population and Global Health, McGill University, Montreal, Canada; 9 Department of Psychiatry and Behavioural Sciences, Punjab Medical College, Faisalabad, Pakistan; 10 HIV Edmonton, Edmonton, Canada; 11 Women in Global Health, Kenya Chapter, Kenya; PLOS: Public Library of Science, UNITED STATES; McGill University, CANADA

There is a growing and overdue recognition in recent years of visa and passport inequities as a significant barrier to global health education, practice, and participation. These inequities largely impact low- and middle-income country (LMIC) citizens, refugees, and asylum seekers, as highlighted by us, other affected individuals, and advocates for systemic change [1–3]. Examples illustrate a range of challenges including structurally discriminatory border control policies by high income countries (HICs), poor administrative due diligence in visa processes, lack of professionalism and inconsistency in assessment procedures during visa interviews or at customs, disregard for extended visa processing timelines by event organizers, associated high costs and ignorance of visa challenges by event organizers, academic institutions, and organizations [3–5]. While highest level of restrictions are imposed by countries in the Global North, it is crucial to note that visa and passport discrimination against LMIC citizens are practiced by countries in the Global South as well [6, 7].

Persisting racist, colonial and neo-colonial ideologies, and political and economic realities impact visa and passport inequities perpetuating a vicious cycle by ‘othering’ LMIC citizens and denying them opportunities to contribute towards achieving the goal of ‘health for all’ [1, 8]. These inequities continue to affect access to global health education, sharing of expertise, participating in conferences, research collaborations, and building professional networks.

Early career scholars, faculty and students face added layers of barriers and consequences due to lack of resources and institutional power [5, 9]. Additionally, these inequities are normalized by the continuation of discriminatory systems and inaction of those with power and privilege [2, 5]. However, the current discourse provides us with a window of opportunity for change. We, as a group of young global health practitioners and trainees with diverse citizenship impacted by these challenges, offer a suite of options to reform current practices and reimagine ways to minimize visa and passport inequities. Recognizing the need for urgent action and the complexity of the challenge we outline short, medium, and long-term solutions.

## Working towards solutions—Short, medium and long term

**Fig 1:**
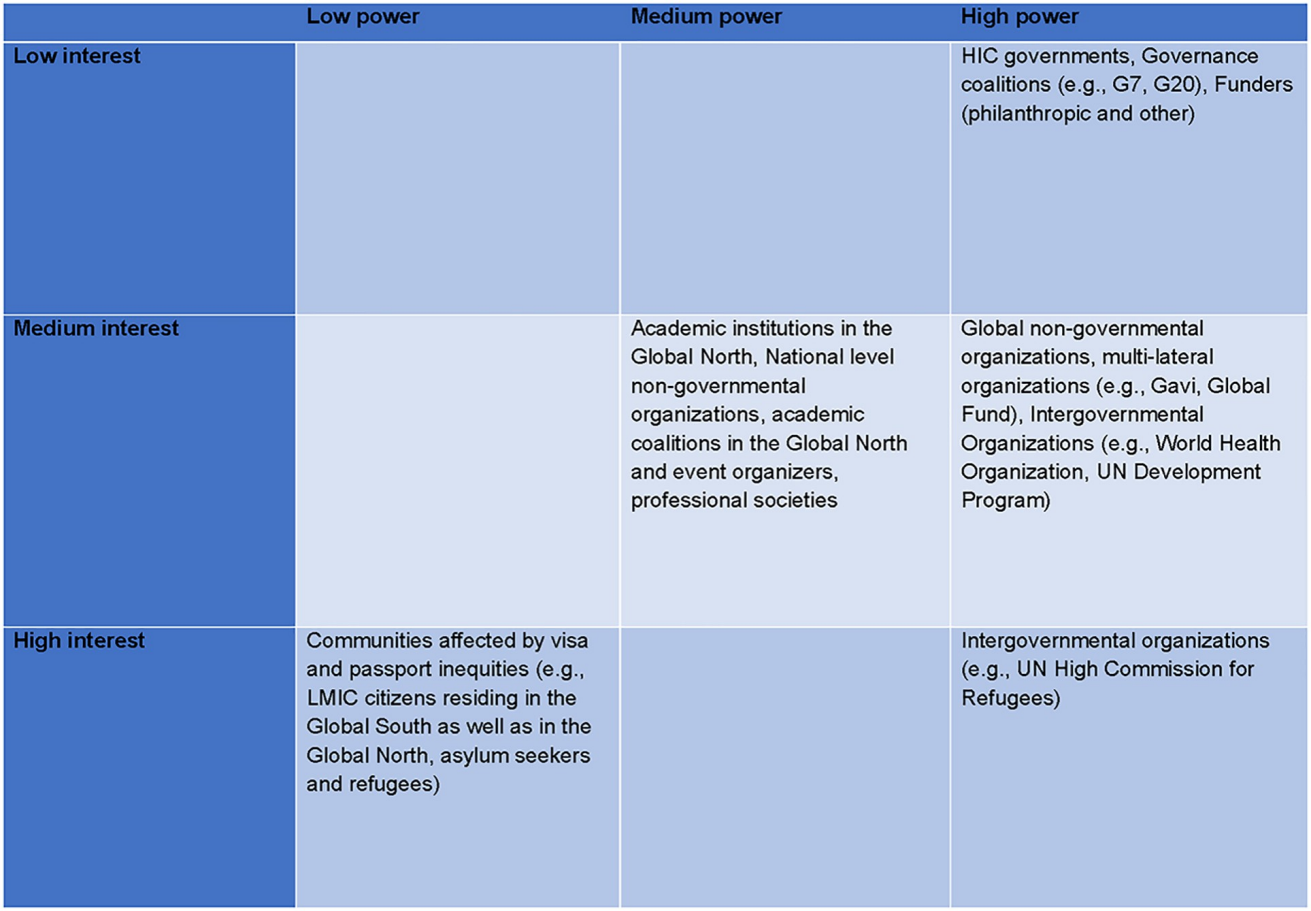
Power and interest matrix among stakeholders relevant to visa and passport inequities. Legend for the matrix in Fig 1: **Power**: The political, institutional and financial power to reduce visa and passport inequities; **Interest**: Perceived interest to reduce visa and passport inequities based on public advocacy efforts.

When addressing visa and passport inequities as a structural problem, it is crucial recognize the power imbalance among stakeholders. In Fig 1, we map key stakeholders, their level of power and perceived level of interest in reducing visa and passport inequities, based on the current advocacy landscape. Recognizing the power imbalance also allows us to understand the reasoning behind calls for collective advocacy and why lending institutional power to address these inequities has instrumental value.

Immediate, short-term considerations to minimize visa and passport inequities start with better planning of global health events. To address visa and passport challenges associated with global health events we suggest a three-pronged approach.

First is having built-in support options as part of the planning and governance processes of global health events to account for visa and passport inequities. These support options include having dedicated teams for timely visa related document provision and coordination, accounting for visa related delays in planning, providing additional funds for participants from the Global South for visa costs and travel expenses, and more affordable registration fees for LMICs and affected communities/advocates. Global health event organizers can proactively inform their governments about major conferences and request for an efficient handling of conference visas.

Second is to choose venues in countries that have minimal visa restrictions. For recurring (e.g., annual) conferences, rotating venues in global locations with low visa restrictions can level the playing field. Making geographic rotation of conferences the norm and budgeting accordingly will be a crucial step in changing the landscape of access. It will also send a clear message that it is no longer acceptable to only host meetings in Global North venues. Organizers can use resources such as https://visaopenness.org to assess the level of visa restrictions. Examples of successful rotation include Women Leaders in Global Health Conference moving from US and UK to Rwanda and the recent announcement by International AIDS Society to rotate conference venues as a response to visa inequities faced by participants [10–12].

Third is effective and judicious use of remote participation technology to facilitate participation. An important qualification here is that the use of technology such as video conferencing should not replace efforts to ensure in-person participation of LMIC experts or to create a two-tier conference where HIC attendees get all the time and attention, while LMICs folks are muted participants with little ability to speak or contribute. However, remote/hybrid options can help experts residing in LMICs with demanding schedules to have better options to consider when joining conference panels than flying over 20 hours or paying a lot of money to attend a two-day conference.

In the medium-term, conference organizers, academic institutes, non-governmental organizations, think tanks and funders need to make visa and passport inequities a priority within their advocacy agendas. This means setting a clear and purposeful agenda to take initiative within their own institutions and governments and to advocate for change in policy arenas without relegating this challenge as an untouchable or unsolvable problem.

Global health departments and schools need to become intentional about educating faculty and administration on visa and passport inequities including discussing solution building in routine meetings. Dedicated support efforts for visas after offering admission, research and conference participation, and partnership building is necessary if we want to achieve genuine global bi-directional knowledge flow [13]. Supportive efforts by institutes can include engaging with relevant ministries, embassies, prioritizing early essential document provision for students, faculty or scholars, providing administrative guidance within school systems and being available to troubleshoot visa processes with students, and establishing special admissions and funding pathways for scholars at risk. There is a growing number of global health institutions setting examples via action such as the McMaster University’s Students at Risk Bursary program, Johns Hopkins Bloomberg School of Public Health scholarships for Syrian refugees, and Scholars At Risk Program at UCSF Institute for Global Health Sciences. Additionally, institutions such as American University of Beirut provide broad support for refugee education. We call on all universities to establish special tracks for accepting and funding forcibly displaced scholars.

Long-term investments for a sustainable, structural shift to solve visa and passport inequities need to be at governance levels–global, national, and organizational. Stakeholders include foreign ministries or embassies, policy makers, multilateral organizations, intergovernmental organizations and global non-governmental organizations with political capital. Meaningful discourse should be a starting point with action-oriented agenda setting as a priority, recognizing the relevance of visa and passport discrimination to global goals. For meaningful discourses to occur, there must be recognition of unfair stereotyping, racism, neo-colonial global structural values, and ignorance of these challenges as HICs are often unaffected by them. Additionally, we need to recognize that often far-right political parties promote anti-immigrant, anti-refugee sentiments pushing for othering of these communities. Thus, citizens have the power to reduce visa discrimination by voting in leaders who will enact real governance reform. There is consistent evidence that visa processes can be alienating, humiliating and opaque processes for citizens of LMICs and other affected individuals [2, 3, 5, 6, 14]. Therefore, training on cultural safety, anti-prejudice and anti-racism, better transparency, context specific timelines such as not delaying visas for conferences or global meetings can be starting points in changing these governance related barriers. Visa and passport inequities are a challenge for advancement of global health and need to be recognized at forums such as the UN General Assembly, World Health Assembly, World Health Summit, Consortium of Universities for Global Health, and other high-level meetings.

Addressing visa and passport inequities is a multi-stakeholder effort, and the burden should not be put on Global South communities to do all the work. In a space where those affected cannot often speak truth to power due to repercussions that might cause harm or stifle their ability to pursue global opportunities, allyship by key powerful Global North stakeholders including academic institutions and global organizations remain vital. If we are to genuinely address visa and passport discrimination, we also need to commit to a paradigm shift within ourselves that resists the othering of people in the Global South.
